# IL-12 DNA Displays Efficient Adjuvant Effects Improving Immunogenicity of Ag85A in DNA Prime/MVA Boost Immunizations

**DOI:** 10.3389/fcimb.2020.581812

**Published:** 2020-09-23

**Authors:** María Paula Morelli, María Paula Del Medico Zajac, Joaquín Miguel Pellegrini, Nicolás Oscar Amiano, Nancy Liliana Tateosian, Gabriela Calamante, María Magdalena Gherardi, Verónica Edith García

**Affiliations:** ^1^Instituto de Química Biológica de la Facultad de Ciencias Exactas y Naturales (IQUIBICEN), Universidad de Buenos Aires (UBA)-Consejo Nacional de Investigaciones Científicas y Técnicas, Buenos Aires, Argentina; ^2^Departamento de Química Biológica, Facultad de Ciencias Exactas y Naturales (FCEN), Universidad de Buenos Aires, Buenos Aires, Argentina; ^3^Instituto de Agrobiotecnología y Biología Molecular (IABIMO), Instituto Nacional de Tecnología Agropecuaria (INTA)-Consejo Nacional de Investigaciones Científicas y Técnicas, Buenos Aires, Argentina; ^4^Instituto de Investigaciones Biomédicas en Retrovirus y SIDA (INBIRS), Facultad de Medicina, Universidad de Buenos Aires-Consejo Nacional de Investigaciones Científicas y Técnicas, Buenos Aires, Argentina

**Keywords:** Ag85A, DNA vaccine, IL-12, MVA, tuberculosis, vaccine

## Abstract

*Mycobacterium tuberculosis* (*Mtb*) infection is one of the leading causes of death worldwide. The Modified Vaccinia Ankara (MVA) vaccine vector expressing the mycobacterial antigen 85A (MVA85A) was demonstrated to be safe, although it did not improve BCG efficacy, denoting the need to search for improved tuberculosis vaccines. In this work, we investigated the effect of IL-12 DNA -as an adjuvant- on an Ag85A DNA prime/MVA85A boost vaccination regimen. We evaluated the immune response profile elicited in mice and the protection conferred against intratracheal *Mtb* H37Rv challenge. We observed that the immunization scheme including DNA-A85A+DNA-IL-12/MVA85A induced a strong IFN-γ production to Ag85A *in vitro*, with a significant expansion of IFN-γ^+^CD4^+^ and IFN-γ^+^CD8^+^ anti-Ag85A lymphocytes. Furthermore, we also detected a significant increase in the proportion of specific CD8^+^CD107^+^ T cells against Ag85A. Additionally, inclusion of IL-12 DNA in the DNA-A85A/MVA85A vaccine scheme induced a marked augment in anti-Ag85A IgG levels. Interestingly, after 30 days of infection with *Mtb* H37Rv, DNA-A85A+DNA-IL-12/MVA85A vaccinated mice displayed a significant reduction in lung bacterial burden. Together, our findings suggest that IL-12 DNA might be useful as a molecular adjuvant in an Ag85A DNA/MVA prime-boost vaccine against *Mtb* infection.

## Introduction

Tuberculosis (TB) continues to be one of the main global health problems. In fact, despite the excellent progress of the Directly Observed Treatment (DOT) strategy, and affordable and effective anti-TB treatments, the World Health Organization estimated 1.45 million deaths due to *Mycobacterium tuberculosis* (*Mtb*) infection in 2018 (World Health Organization, 2019). TB has always been correlated with poverty, especially in low- and middle-income countries. However, the incidence of TB has increased even in industrialized countries, mainly in vulnerable groups. Furthermore, the strategies for TB control have been hampered by the Human Immunodeficiency Virus (HIV) co-infection and the emergence of drug-resistant *Mtb* strains. Therefore, developing a vaccine capable of preventing *Mtb* infection would contribute to controlling the TB epidemic.

The attenuated *Mycobacterium bovis* Bacillus Calmette-Guérin (BCG) has been the only vaccine available to be used in humans for almost 100 years. BCG is effective in protecting against pulmonary and extrapulmonary TB in children up to 10 years old (Abubakar et al., 2013), but protection against the pulmonary form of TB in adults remains highly controversial (Hatherill et al., 2020).

Currently, there are 15 vaccines in clinical trial phase I, II, or III (Home—ClinicalTrials.gov^1^). The promising vaccine Modified Vaccinia Ankara (MVA) vector expressing the mycobacterial antigen 85A (MVA85A) was particularly well-tolerated in HIV-1 infected adults (Ndiaye et al., 2015) and infants (Tameris et al., 2013). Unfortunately, the BCG/MVA85A immunization scheme failed to provide protection in phase IIb clinical trials including both neonates and adults (Tameris et al., 2013; Ndiaye et al., 2015). One possibility regarding the low efficacy of the MVA85A might be the restrictive replication capacity of the MVA (Gilbert, 2013). Several strategies have been explored in order to augment MVA immunogenicity. Some of those strategies include heterologous prime/boost protocols, the use of co-stimulatory molecules, the deletion of viral immunomodulatory genes still present in the poxvirus genome and the combined use of adjuvants (García-Arriaza and Esteban, 2014). Therefore, protocols that enhance the immune response induced by MVA85A might be useful to improve the protection of TB vaccines.

Interleukin-12 (IL-12) is a heterodimeric cytokine that plays an essential role in cellular and humoral immunity. Indeed, IL-12 constitutes an important link between innate and adaptive immunity (Gately et al., 1998; Trinchieri, 2003). IL-12 is a pro-inflammatory cytokine produced by antigen-presenting cells like dendritic cells (DCs) and macrophages. IL-12 regulates T-cell and natural killer (NK) cell responses. The importance of IL-12 during the host immune response against *Mtb* infection has been demonstrated both experimentally and by the increased susceptibility of humans deficient in the IL-12 pathway (Trinchieri, 2003). Moreover, the use of IL-12 as an adjuvant expressed from a plasmid vector in heterologous schemes has been successfully explored (Gherardi et al., 2000, 2001, 2003; Abaitua et al., 2006; Rodríguez et al., 2012; Kalams et al., 2013; Maeto et al., 2014; Elizaga et al., 2018). Additionally, the immune modulator role of IL-12 DNA plasmid when included in DNA vaccines against *Leishmania major* (Tapia et al., 2003) and Hepatitis B Virus (Chow et al., 1998; Du et al., 2003) infections, has been also evaluated (Hanlon et al., 2001). However, so far, IL-12 has been scarcely used as an adjuvant in the context of TB vaccines.

In the present work, we investigated the use of IL-12 as a molecular adjuvant included in a particular DNA prime/MVA boost vaccine schedule. Our findings indicate that IL-12 DNA might be used to increase the host immune response generated by MVA85A against *Mtb* infection.

## Materials and Methods

### DNA Vaccines

The plasmid pCI-neo (Promega) carrying the coding sequence of Ag85A (pCI-Ag85A) was previously obtained at Dr. Calamante's laboratory at the IABIMO. The expression of Ag85A was confirmed by Western blot using total protein extracts from Baby hamster kidney (BHK-21) transfected cells and a polyclonal anti-Ag85A antibody (Supplementary Figure 1).

The DNA plasmid expressing murine IL-12 (carrying the p35 and p40 murine genes, pCI-IL12) (Gherardi et al., 2000; Tapia et al., 2003) was kindly provided by Dr. Mariano Esteban [Department of Molecular and Cellular Biology, National Center of Biotechnology (CNB), Superior Council of Scientific Investigations (CSIC), Madrid, Spain]. The correct expression of the cytokine from the plasmid pCI-IL12 was corroborated by quantifying IL-12 release in transfected cells' supernatants. Briefly, mouse 3T3 cells were transfected with 5 μg of DNA-IL-12, using Lipofectamine (Invitrogen) according to the manufacturer's instructions. Forty-eight hours post-transfection, 1.8 × 10^5^ pg/ml of IL-12 were detected by ELISA (optEIA, BD) (Maeto, 2015).

Plasmids were purified from *Escherichia coli* DH5α with Endo free Maxi-Prep purification kits (NucleoBond Xtra Maxi Plus EF, Macherey-Nalgen) using pyrogen-free material.

### Viruses

MVA85A encoding the complete sequence of Ag85A was previously obtained at Dr. Calamante's laboratory at IABIMO. Viral stocks of MVA (expressing ovalbumin, an irrelevant protein that does not overlap with Ag85A) and recombinant MVA85A were propagated in chicken embryo fibroblasts, clarified and titrated as described elsewhere (Cotter et al., 2017). The expression of Ag85A was assessed by Western blot using total protein extracts of chicken embryo fibroblasts infected with MVA85A and a polyclonal anti-Ag85A antibody (Supplementary Figure 1).

### Antigen

*In vitro* stimulation of cells was performed with recombinant Ag85A (BEI Resources, NIAID, NIH: Ag85A, Recombinant Protein Reference Standard, NR-49427) resuspended in phosphate-buffered saline (PBS; 1.37 mM NaCl, 27 mM KCl, 2 mM KH_2_PO_4_, 80 mM Na_2_H_2_PO_4_ anhydrous in water).

### Animals and Immunization

Six- to eight-week-old, pathogen-free BALB/c female mice were purchased from the Central animal facility at the University of Buenos Aires, School of Sciences. Animals were housed in biosafety level 3 animal facilities at the Operative Unit of the Biological Containment Center from the National Administration of Laboratories and Health Institutes “Dr. Carlos G. Malbrán” (UOCCCB-ANLIS), according to approved proceedings (Approval code: DEP-20-00). Mice were kept on ventilated racks and fed a standard *ad libitum* diet. All experimental work was performed following International Guidelines (European Parliament European Council, 2010; National Research Council, 2011). All efforts were made to minimize suffering of the animals.

Mice (4–5/group) received three intramuscular (i.m.) administrations (separated by 2-week intervals) of plasmid DNA (100 μg each time). Two weeks after the last immunization, mice were intraperitoneally (i.p.) inoculated with 10^7^ plaque-forming units (PFU) of MVA. DNA vaccine vectors were diluted in sterile PBS (final volume 50 μl). pCI-IL12 was administered in conjunction with pCI-Ag85A. Sera and splenocytes were collected 8 days after the last immunization. An empty pCI plasmid was used as a DNA control. A recombinant MVA expressing an irrelevant protein was employed as MVA control.

Mice were anesthetized, and blood samples were obtained by cardiac puncture. Then blood was allowed to clot (4 h at room temperature), centrifuged and sera were collected and stored at −80°C.

Splenocytes were isolated by routine methods. Briefly, the spleens were gently disrupted through a 40-μm cell-strainer (BD Biosciences). Subsequently, red blood cells were lysed, and splenocytes were washed with RPMI (RPMI-1640 medium, Gibco BRL) plus 5% (v/v) fetal bovine serum (FBS, Natocor). Splenocytes were then resuspended at 10 × 10^6^ cells/mL in RPMIc [RPMI-1640 medium (Gibco BRL) supplemented with 2 mM L-glutamine (Gibco BRL), 100 U/mL penicillin (Gibco BRL), 0.1 mg/mL streptomycin (Gibco BRL), 10 mM HEPES (Gibco BRL), and 0.055 mM β-Mercaptoethanol (Gibco BRL), with 10% (v/v) FBS (Natocor)].

### Intracellular Cytokine Staining (ICS)

Splenocytes were cultured (10^6^ cells/wells) in 96-well U bottom plates with RPMIc for 16 h, with or without Ag85A protein (5 μg/mL) plus anti-CD28 (1 ng/mL; BD Biosciences). In some experiments, anti-CD107a and anti-CD107b (CD107a/b-FITC; BD Biosciences) antibodies were added to Ag85A stimulated cells to measure the cytotoxic activity of CD8 T lymphocytes (Betts et al., 2003). Cells stimulated during 5 h with phorbol myristate acetate (PMA, 10 ng/mL, Sigma-Aldrich) plus ionomycin (250 ng/mL, Sigma-Aldrich) were used as positive controls. Monensin (2 μM; Sigma-Aldrich) was added for the last 5 h of culture. After washing, anti-CD4-APC and anti-CD8-PerCP (BioLegend) antibodies were added for 30 min. Then, cells were fixed using 2% paraformaldehyde (PFA) and permeabilizated with 0.5% (v/v) saponin (Sigma-Aldrich), 0.01% (w/v) sodium azide, and 10% (v/v) FBS (Natocor) in PBS. Finally, cells were stained using anti-IFN-γ-PE (Biolegend) for 30 min at 4°C in the dark. Cells were acquired on a FACSAria II flow cytometer (BD Biosciences). Isotype matched controls and fluorescence minus one (FMO) controls were included in each experiment. Data were analyzed using FlowJo v10 software (BD Biosciences). Gates strategies and FACS examples are shown in **Figure 3**.

### Cytokine Production

Splenocytes were cultured at 10^6^ cells/well (in triplicate wells) in 96-well U bottom plates with RPMIc for 72 h with or without recombinant Ag85A (5 μg/mL). Cells stimulated with Concanavalin A (ConA, 1 mg/mL) or PBS were used as positive and negative controls, respectively. Culture supernatants were stored at −80°C until assayed. IFN-γ analysis was performed using ELISA BD OptEIA set (BD Biosciences), according to the manufacturer's instructions.

### Antibody Measurement

The presence of serum anti-Ag85A Abs was assayed by ELISA. Ninety-six-well plates were coated with purified Ag85A at 2.5 μg/mL. Blockage was performed with 10% (v/v) FBS in PBS-0.05% Tween 20. Samples (duplicates) were diluted in blocking solution. Detection was performed using mouse anti-IgG conjugated to peroxidase enzyme (Jackson Immuno Research). TMB Peroxidase substrate (Sigma-Aldrich) was added and the reaction was stopped by adding 2 N H_2_SO_4_. Absorbance was measured at 450 nm.

### *M. tuberculosis* Challenge Infection

*Mycobacterium tuberculosis* H37Rv strain (*Mtb* H37Rv) was grown in Middlebrook 7H9 broth (BD Biosciences) or in 7H10 agar (BD Biosciences), 0.2% (v/v) glycerol, and albumin-dextrose-catalase-oleic acid supplement (BD Biosciences). Cultures were harvested at exponential growing phase at 37°C and clumps were disaggregated by glass bead vortexing and passage through a 25 Gauge needle. Bacteria were centrifuged twice for 10 min at 3,300 × g and supernatants were diluted with physiological solution. Finally, OD at 600 nm was determined. *Mtb* H37Rv bacterial growth was performed in BSL3 security cabinets at the UOCCCB-ANLIS.

Groups of five female BALB/c mice were intratracheally inoculated with *Mtb* H37Rv (1 × 10^4^ colony-forming unit (CFU)/mice) as previously described (Revelli et al., 2012). Briefly, animals were anesthetized with a mixture of ketamine and xylazine (100 and 10 mg/kg, respectively), placed in supine position over an acrylic backboard and the inoculum (20 μL) was injected through the trachea with a Hamilton syringe coupled to a blunt-ended probe. Mice were euthanized at 30 days post-infection by an intraperitoneal injection of a lethal dose of ketamine and xylazine, and their spleens and lungs were aseptically removed. The whole organs were homogenized in 2 mL of sterile RPMI. Then, triplicates of serial dilutions were plated in Middlebrook 7H10 agar for CFU counting.

### Data Analysis

The quantitative data were expressed as mean ± standard error of the mean (SEM). Analysis of variances (ANOVA) followed by Holm-Sidak's multiple comparison tests were used to analyze differences between groups. For categorical variables, the Fisher exact test was performed to compare proportions of infected spleens. GraphPad Prism 6.0 (GraphPad Software) was used for all statistical analysis. A *p* < 0.05 was considered as statistically significant.

## Results

**Figure 1:**
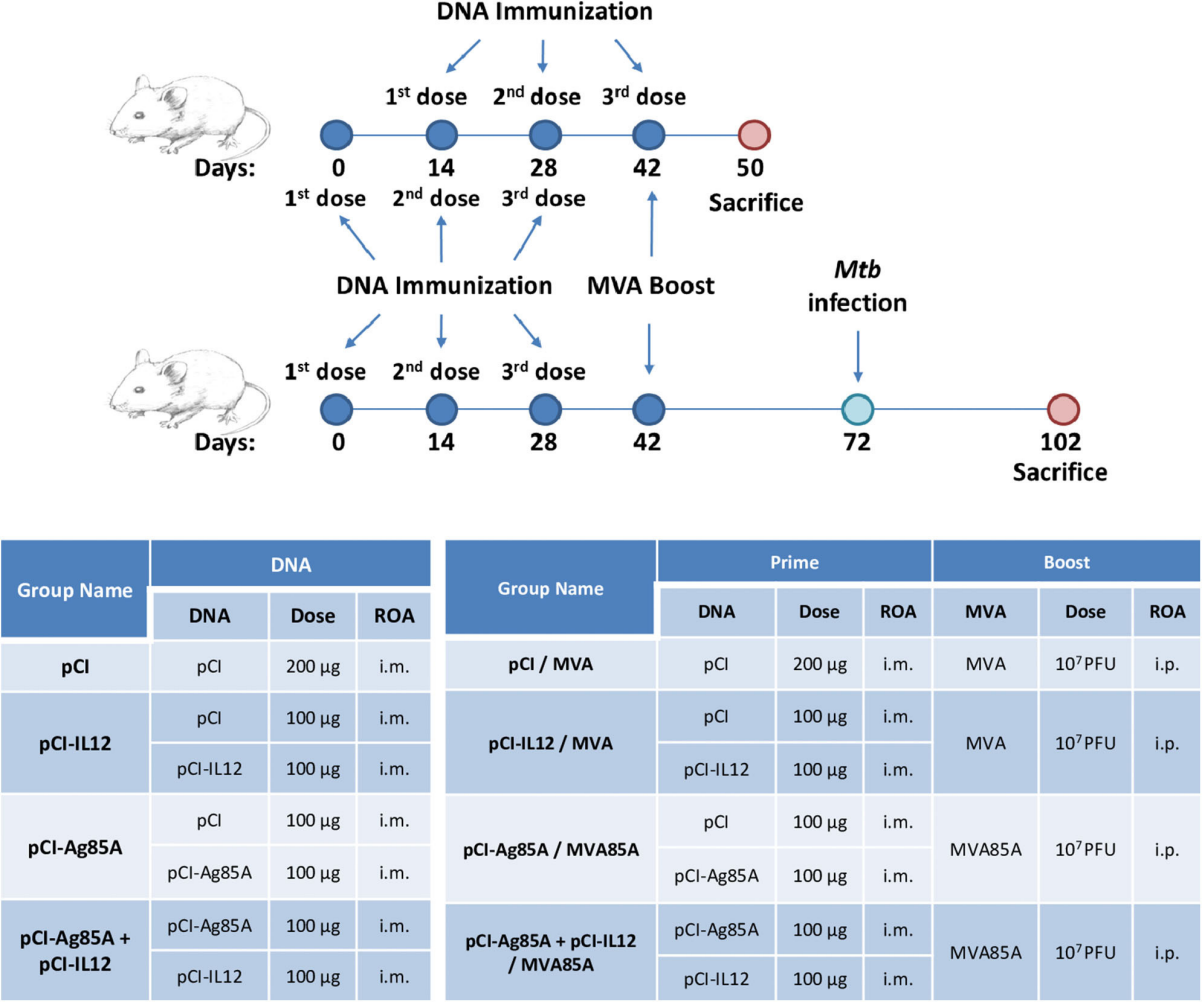
Experimental design. ROA, Route of Administration; i.m., intramuscular; i.p., intraperitoneally; pCI, pCI empty; pCI-IL12, pCI expressing IL-12; pCI-Ag85A, pCI expressing Ag85A; MVA, recombinant MVA expressing an irrelevant protein; MVA85A, recombinant MVA expressing Ag85A.

In order to evaluate the potential adjuvant role of IL-12 (expressed from a DNA vector) to increase the efficacy of pCI-Ag85A and the heterologous pCI-Ag85A/MVA85A immunization scheme, we immunized 6- to 8-week-old BALB/c mice as shown in Figure 1. Briefly, each group of animals was inoculated by the i.m. route with three doses of DNA vectors separated by 2 weeks among them. In some groups, after 14 days of the last priming dose, mice were i.p. boosted with 10^7^ PFU/animal of MVA85A. Mice from control groups were immunized with empty DNA plasmid (pCI) and a recombinant MVA expressing an irrelevant protein. Eight days after the last dose, we analyzed the humoral and cellular immune responses against Ag85A elicited by animals from the different vaccinated groups.

**Figure 2:**
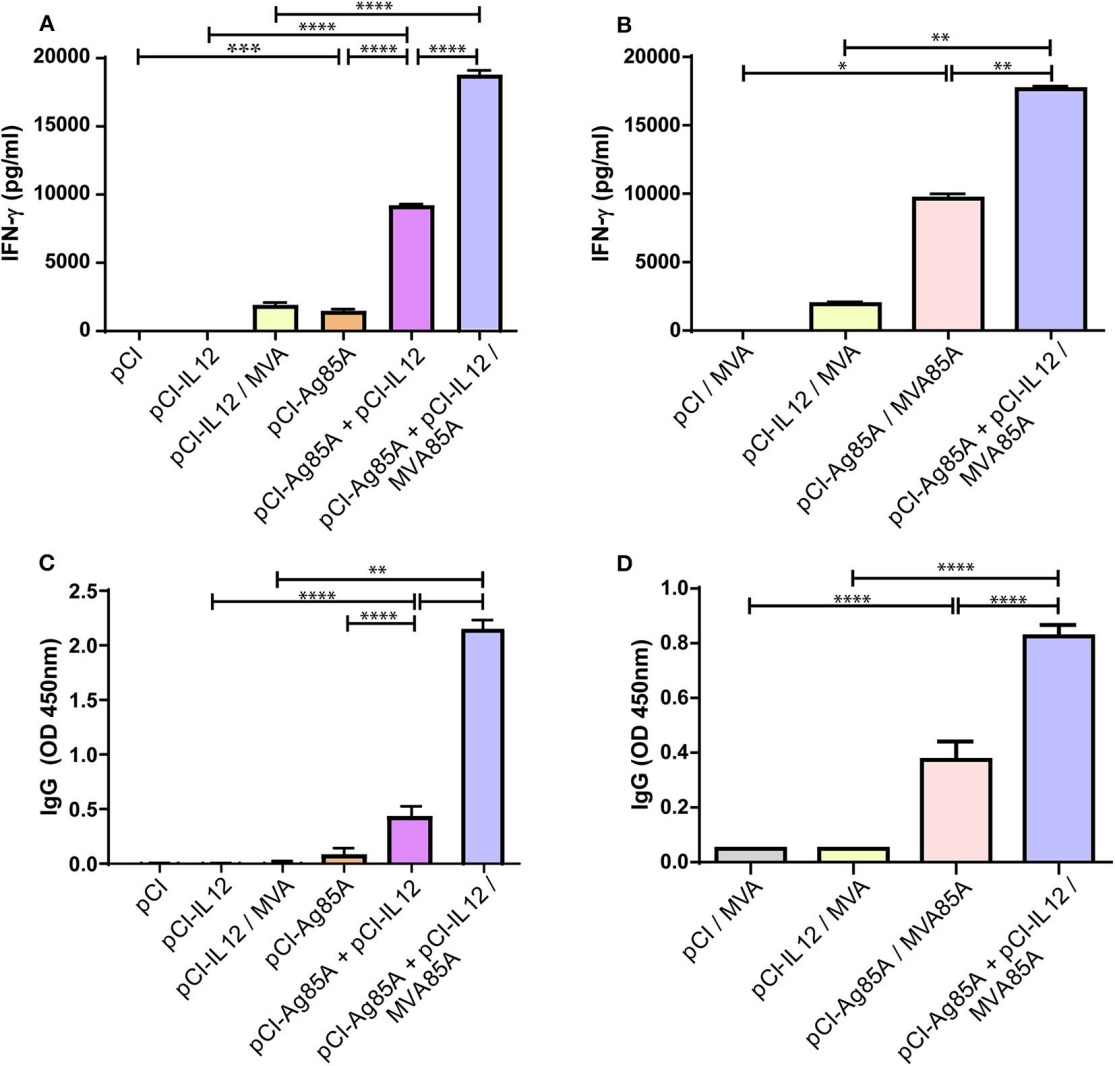
IL-12 DNA administration enhances the immune response of the host to Ag85A. **(A,B)** Splenocytes were stimulated *in vitro* with Ag85A (5 μg/mL), ConA or PBS for 72 h. IFN-γ production was then determined by ELISA. Each bar represents the mean of IFN-γ production (PBS subtracted) ± SEM. **(C,D)** Anti-Ag85A IgG levels in mice serum were determined by ELISA. The samples were diluted **(C)** 1/100 and **(D)** 1/10,000 in PBS-10% fetal bovine serum. Each bar represents the mean of OD 450nm ± SEM. Comparisons among groups were determined by one-way ANOVA followed by Holm-Sidak's multiple comparison test. A value of *p* < 0.05 was considered statistically significant. *p < 0.05, **p < 0.01, ***p < 0.001, and ****p < 0.0001. Results are representative of two independent experiments.

To characterize the host cellular immune response generated by vaccination during the acute phase, we isolated splenocytes 8 days after the last inoculation. Cells were then stimulated *in vitro* with Ag85A, PBS (negative control), or ConA (positive control) for 72 h. Considering that a type 1 immune response generated after vaccination is critical for protection against *Mtb*, we analyzed IFN-γ levels in culture supernatants (Figures 2A,B). Mice immunized with pCI-Ag85A expressed low levels of IFN-γ. However, a significant increase in the amount of this cytokine was detected when pCI-IL12 was co-administered to the animals. Furthermore, the highest levels of IFN-γ were observed in mice that were boosted with MVA85A (Figure 2A). As expected, splenocytes from pCI-Ag85A, pCI-Ag85A+pCI-IL12, pCI-Ag85A/MVA85A, and pCI-Ag85A+pCI-IL12/MVA85A mice produced significantly higher IFN-γ levels in response to Ag85A stimulation as compared to their controls. Interestingly, co-administration of pCI-IL12 and pCI-Ag85A significantly augmented the levels of IFN-γ, denoting the clear adjuvant effect of IL-12 (Figure 2B).

Although the relevance of antibodies in the protection against *Mtb* is not clear, the humoral response cannot be ruled out as a factor that contributes to *Mtb* containment. Protective antibodies might promote and increase intracellular bacterial death through phagocytosis mediated by FcR, and improve the immune response via the rapid antigen adsorption and processing. Therefore, we evaluated the levels of anti-Ag85A IgG in serum. As shown in Figure 2C, we observed a slight increment of anti-Ag85A IgG levels when pCI-IL12 was co-administered with pCI-Ag85A. Nevertheless, MVA85A boost induced a significant increase in anti-Ag85A IgG in serum of vaccinated mice. Importantly, when we evaluated the effect of IL-12 on the MVA boosted strategy, mice that received pCI-IL12 as an adjuvant displayed the highest levels of anti-Ag85A IgG (Figure 2D).

**Figure 3:**
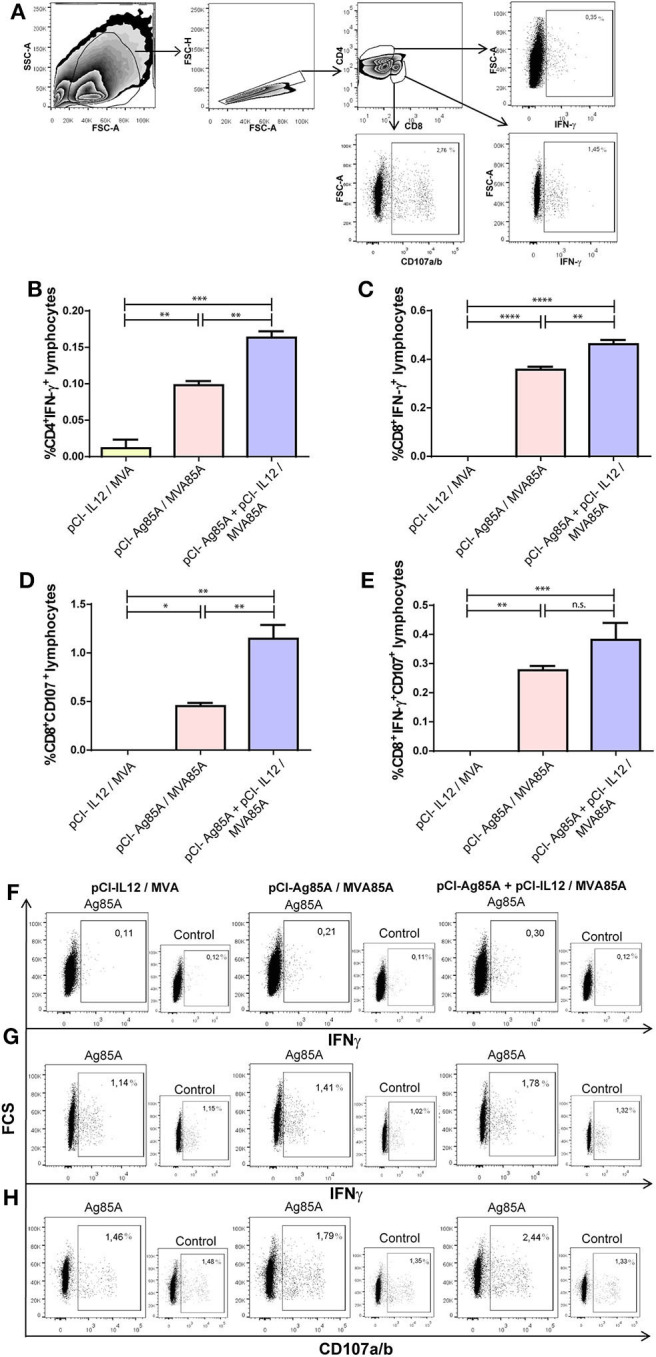
The co-administration of IL-12 DNA induced a strong cellular immune response against the Ag85A in mice spleen. Splenocytes from immunized mice were stimulated *in vitro* with PBS or recombinant Ag85A for 16 h. Then, IFN-γ production and CD107a/b surface expression were determined by flow cytometry. **(A)** Gate strategy. CD4^+^ or CD8^+^ IFN-γ^+^ T cells and CD8^+^CD107a/b^+^ T lymphocytes were identified by flow cytometry first gating on lymphocytes by light scatter; then, gating on CD4^+^ and CD8^+^ T cells; and finally, gating according to IFN-γ^+^ and CD107a/b^+^ expression using the AND boolean gate tool from FlowJo v10 software. Each bar represents the mean (PBS subtracted) of the **(B)** %CD4^+^ IFN-γ^+^ cells ± SEM, **(C)** %CD8^+^IFN-γ^+^ cells ± SEM, **(D)** %CD8^+^CD107a/b^+^ cells ± SEM, **(E)** %CD8^+^CD107a/b^+^IFN-γ^+^ cells ± SEM. Comparisons among groups were determined by one-way ANOVA followed by Holm-Sidak's multiple comparison test. A value of *p* < 0.05 was considered statistically significant. n.s., non-significant difference, *p < 0.05, **p < 0.01, ***p < 0.001, and ****p < 0.0001. Results are representative of two independent experiments. Representative dot plots are shown for **(F)** % CD4^+^IFN-γ^+^ T cells, **(G)** % CD8^+^IFN-γ^+^ T cells, and **(H)** % CD8^+^CD107a/b^+^ T lymphocytes (large quadrants). Controls (small quadrants) are shown.

Next, we investigated whether the use of IL-12 as an adjuvant stimulated the production of IFN-γ by different populations of T lymphocytes. To do this, 8 days after the last antigenic dose, mice were sacrificed, and recovered splenocytes were stimulated *in vitro* with Ag85A. We observed that pCI-Ag85A/MVA85A immunization induced a marked augment in the percentage of CD4^+^ and CD8^+^ IFN-γ^+^ T lymphocytes (Figures 3B,C). Even more, when pCl-IL12 was co-administrated with pCI-Ag85A, a significantly higher increase in the percentage of CD4^+^IFN-γ^+^ and CD8^+^ IFN-γ^+^ T cells was detected as compared to the pCI-Ag85A animal group (Figures 3B,C).

Besides, in order to evaluate the cytotoxic potential of specific CD8^+^ T cells expanded by the immunization, we incubated splenocytes from the different immunized groups with Ag85A plus anti-CD107 monoclonal antibody. As shown in Figure 3D, pCI-Ag85A/MVA85A immunization induced the expression of CD107a/b on CD8^+^ T lymphocytes. Importantly, this response resulted significantly improved when IL-12 was included in the immunization scheme (Figure 3D). Even more, the adjuvant effect of IL-12 was also observed when the percentage of CD8^+^IFN-γ^+^CD107a/b^+^ cells induced in immunized mice was analyzed (Figure 3E). In conclusion, these results denote that additional benefits of including IL-12 adjuvant in vaccine formulations may be the expansion and activation of CD8^+^ cytotoxic T lymphocytes.

**Figure 4:**
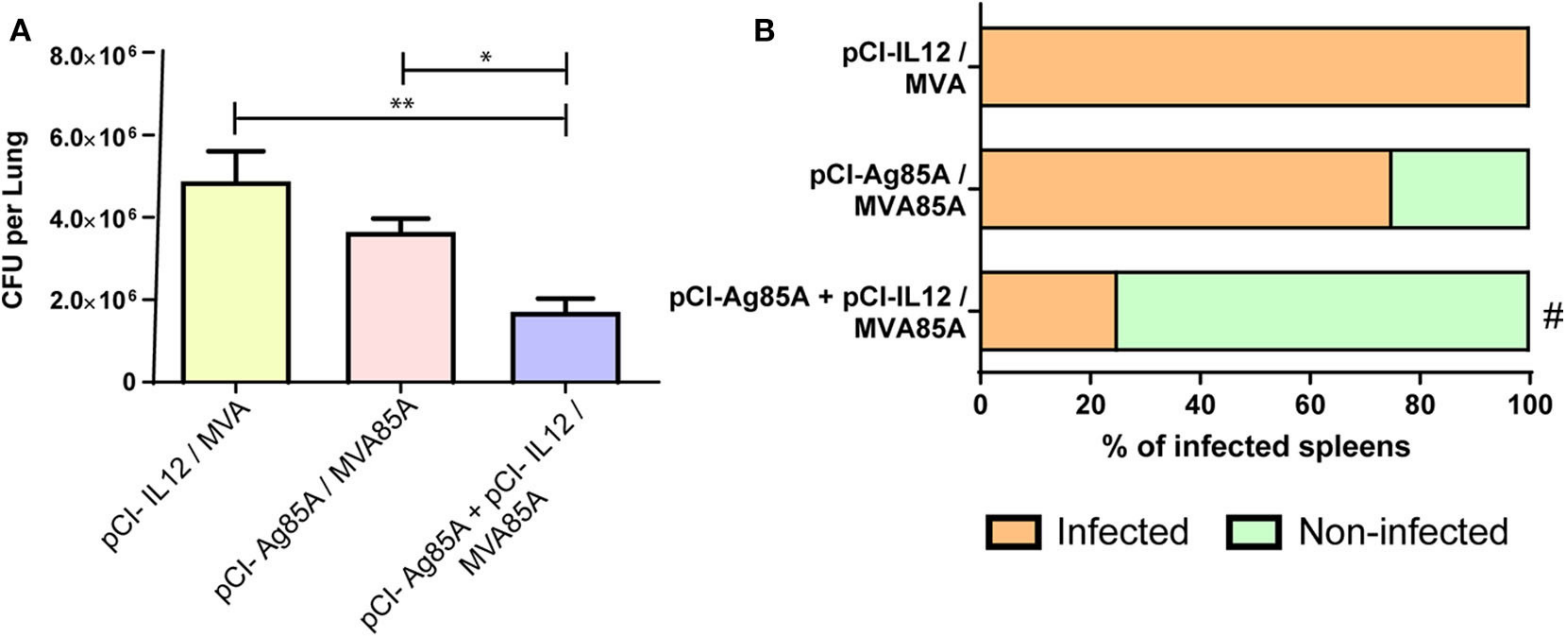
pCI-IL12 improves control of *Mtb* H37Rv in mice. Immunizations were performed as described above. Four weeks after the last immunization, mice were challenged with *Mtb* H37Rv via intratracheal route (*n* = 4–5/group). Mice were sacrificed 4 weeks after challenge. **(A)** CFUs in lungs were analyzed by culturing lung homogenates and enumerating the bacteria. Each bar represent the median (CFU/lung for each group of mice). Comparisons among groups were determined by one-way ANOVA followed by Holm-Sidak's multiple comparison test. A value of *p* < 0.05 was considered statistically significant. *p < 0.05 and **p < 0.01. **(B)** The presence of *Mtb* in spleens was analyzed in all the studied groups. The graph shows the percentage of mice displaying bacteria in their spleens. Comparisons with control groups were determined by Fisher's exact test, ^#^*p* < 0.05. A representative example of three assays is shown.

Considering our findings demonstrating that pCI-IL12 potentiates the anti-Ag85 systemic immune response induced by pCI-85A/MVA85A vaccination, we next decided to evaluate its efficacy against *Mtb* containment. For this, 4 weeks after the last immunization (Figure 1), mice were intratracheally infected with pathogenic *Mtb* H37Rv strain. After 30 days, the number of CFU per lung and the presence of bacteria in spleen were determined. As shown in Figure 4, pCI-Ag85A/MVA85A vaccination slightly diminished the total CFU per lung as compared to control animals. In contrast, by using pCI-IL12 during vaccination, a significant decrease in the number of CFU per lung was observed as compared to animals that did not receive the adjuvant (Figure 4A). In line with these results, 75% of pCI-Ag85A/MVA85A vaccinated mice displayed *Mtb* in their spleens, whereas only 25% of pCI-Ag85A+pCI-IL12/MVA85A vaccinated animals presented bacteria in their spleens (Figure 4B). These results suggest an association between the increase in the cellular immune response and the protection improvement in mice that received IL-12.

## Discussion

Nowadays, *Mycobacterium bovis* BCG displays 90% of effectiveness in childhood tuberculous meningitis and miliary TB (Mangtani et al., 2014). However, regarding pulmonary TB, the most common form of the disease in adults and teenagers (World Health Organization, 2019), BCG confers 59% of protection in children (Mangtani et al., 2014). Furthermore, BCG does not protect after 10–15 years of vaccination in endemic countries (Sterne et al., 1998; Abubakar et al., 2013). Thus, in order to eradicate TB, new vaccines conferring protection during the whole life of the individual are required.

Preventive vaccines against TB can be classified into four broad categories: viable whole-cell vaccines (such as VPM1002, MTBVAC or BCG revaccination), inactivated whole-cell vaccines (such as Vaccae™, RUTI, DAR-901, MIP, or AEC/BCO2), protein subunit vaccine (such as ID93/GLA-SE, H56:IC31, M72/AS01E, or GamTBVac), and viral-vectored vaccines (such as ChAdOx185A-MVA85A, TB/FLU-04L, or Ad5Ag85A) (Home—ClinicalTrials.gov^1^). Among these 15 vaccine candidates in clinical phase, the adjuvants employed include GLA-SE, AS01E, IC31, and CpG plus alum salt-based adjuvant or DEAE-dextran core. All of the mentioned adjuvants are included in protein subunits vaccines formulations, and there are currently no clinical trials reports using DNA vaccines. Given the importance of IL-12 during the immune response of the host against *Mtb* infection, its use as a vaccine adjuvant has been previously proposed (Ha et al., 2006; Yoshida et al., 2006).

IL-12 helps naïve T cells to switch to Th1 lymphocytes and increases T cells cytotoxicity. The use of IL-12 as an adjuvant has been investigated in vaccines against several pathogens (Chow et al., 1998; Gherardi et al., 2000, 2001, 2003; Du et al., 2003; Tapia et al., 2003; Abaitua et al., 2006; Rodríguez et al., 2012; Kalams et al., 2013; Maeto et al., 2014; Elizaga et al., 2018). However, the study of IL-12 as an adjuvant in TB vaccines has been scarcely explored. This might be partly because recombinant IL-12 is quickly removed and inactivated *in vivo* (Ha et al., 2006). In fact, to avoid IL-12 removal, Ha et al. (2006) encapsulated recombinant IL-12 in microspheres to achieve its sustained local expression. The authors showed that the combination of IL-12 plus AS01B adjuvants in a TB subunit vaccine-induced mice immunity and protection against *Mtb* challenge. In addition, Yoshida et al. used the hemagglutinating virus of Japan (HVJ)-liposome method to encapsulate mouse IL-12 expression vector (mIL-12). Then, the authors immunized mice with mIL-12/HVJ plus DNA expressing IgHsp65 (Yoshida et al., 2006). However, no significant differences in the number of CFU per lung were detected between the mIL-12/HVJ (control) and the IgHsp65+mIL-12/HVJ groups (Yoshida et al., 2006). Therefore, the protection described by these authors would not be associated with the host immune response against Hsp65 antigen immunization.

Cytokines are crucial initiators and regulators of the immune response. In TB, key effectors related to host protection include IL-12 and IFN-γ (North and Jung, 2004; Cooper, 2009). IL-12 displays multiple biological functions in immunity modulation, linking the early innate immune response with the subsequent antigen-specific adaptive immunity (Hamza et al., 2010). Accordingly, it has been demonstrated that mice deficient in IL-12 were more susceptible to *Mtb* infection than wild-type animals (Hölscher et al., 2001). Moreover, IL-12 is critical for activation of CD4^+^T cells, leading to the protective Th1 response that promotes IFN-γ secretion by T lymphocytes. Therefore, given the crucial role of IL-12 in TB infection, in this study, we evaluated the incorporation of plasmid DNA encoding murine IL-12 during Ag85A DNA prime followed by a heterologous boost with MVA85A. In line with our work, other authors have previously evaluated the modulation of the response induced by DNA vaccines co-injected with pro-inflammatory cytokines in individual vectors (Kim et al., 1998; Tapia et al., 2003; Rodríguez et al., 2012). Initially, we analyzed the adjuvant effect of IL-12 DNA on Ag85A DNA immunization as compared to mice that received Ag85A DNA alone (Figures 2A,C). We observed that IL-12 DNA co-administration significantly increased both the levels of IgG and IFN-γ in response to Ag85A. Furthermore, when mice received a boost of MVA85A, even higher levels of IgG and IFN-γ against Ag85A were detected (Figure 2). Moreover, the IFN-γ secretion augment was a reflection of the increase of CD4^+^ and CD8^+^ IFN-γ producing lymphocytes (Figures 3B,C). In addition, the co-administration of IL-12 adjuvant induced the expansion of a higher proportion of cytotoxic CD8 T cells (Figures 3D,E). Even more, mice that received a vaccination scheme augmenting the host immune response showed the highest protection against *Mtb* challenge. However, in order to further analyze bacterial load, future studies including staining of *Mtb* (such as acid-fast staining) in tissue samples should be performed.

In this study, we employed Ag85A and IL-12 in different vectors. We had previously used this same strategy using either IL-12 or GM-CSF in trans-expression, with optimal results (Rodríguez et al., 2012). Considering that IL-12 is a soluble mediator required in the microenvironment, the use of individual vectors might have a positive effect without the need to be expressed in the same cell as the antigen. Other authors have used different cytokines expressed by DNA in trans as adjuvants in DNA vaccines (Hanlon et al., 2001; Tapia et al., 2003; Abaitua et al., 2006). However, in the context of TB disease, Sun et al. (2017) have previously employed the cis-expression of Ag85A and IL-15 with encouraging results.

During clinical trials, MVA85A was commonly administered by the intradermal route (Tameris et al., 2013; Ndiaye et al., 2015). Nevertheless, when Tchilian et al. (2009) used the intradermal route to apply MVA85A as a BCG boost in the murine model, they were unable to observe any improved effect beyond the protection conferred by BCG. Furthermore, MVA85A immunogenicity and efficacy in mice have been demonstrated using nasal (Goonetilleke et al., 2003) or intravenous (McShane et al., 2002; Romano et al., 2006) routes. Thus, since immunization with DNA vaccines is usually performed by i.m. route while MVA boost is normally achieved by i.p. route (Rodríguez et al., 2012; Pérez et al., 2020) we used those immunization routes in our mice model. Importantly, this immunization strategy using an adjuvant plus a viral vector was used with the ultimate aim of eliciting efficient humoral and cellular immune responses against the selected antigen.

Several cytokines have a critical function in the immune response of the host against *Mtb* (Domingo-Gonzalez et al., 2016). The central role of IFN-γ in the immune response against *Mtb* infection has been widely demonstrated both in mouse models (Flynn et al., 1993) and humans. Accordingly, we previously reported that TB patients with a weak immune response against *Mtb* and impaired IFN-γ production displayed the most severe disease (Pasquinelli et al., 2004). Moreover, we demonstrated that IFN-γ promotes autophagy in infected monocytes from TB patients contributing to the elimination of *Mtb* (Tateosian et al., 2017). In the present work, we focused on IFN-γ determination, although the role of other crucial cytokines in TB infection should be evaluated in the future. In fact, Sakai *et al*. reported that IFN-γ from CD4^+^ T lymphocytes were responsible for only one third of the total anti-bacterial effects of CD4^+^ T cells in the lungs (Sakai et al., 2016). Furthermore, increasing IFN-γ production led to the early death of the host in mice infection models (Sakai et al., 2016). Additionally, it has been reported that a polyfunctional *Mtb* specific CD4^+^ T cell immune response is required for protective pulmonary immunity during TB (Wu et al., 2017). In fact, IFN-γ could be a part of the response of polyfunctional *Mtb* specific lymphocytes (IFN-γ/IL-2/IL-17/TNF-α) but the role of the other cytokines in the immune response of the host against *Mtb* has to be considered as well. Nevertheless, Beveridge et al. (2007) reported that MVA85A immunization induced polyfunctional CD4^+^ T lymphocytes producing TNF-α, IFN-γ, and IL-2 in humans, but no increase in protection was observed. Moreover, the development of phase I of the AERAS-402 candidate vaccine (a recombinant adenovirus which expresses the *Mtb* antigens Ag85A, Ag85B, and TB10.4) demonstrated that polyfunctional T cell response were not directly associated with the ability of these lymphocytes to identify *Mtb* infected cells (Nyendak et al., 2016). In any case, the role of polyfunctional T cells in our present model should be investigated in future experiments.

The adjuvant potential of IL-12 to induce cytotoxicity has been previously demonstrated (Okada et al., 1997; Tsuji et al., 1997; Belyakov et al., 1998; Tatsumi et al., 2001; Moore et al., 2002; Matsui et al., 2003). *Mtb* can infect epithelial and endothelial cells, and CD8 lymphocytes (together with NK cells) have the ability to eliminate infected cells. Furthermore, cytotoxic CD8 cells have a critical role in killing infected macrophages during TB. Our findings showed that immunization with IL-12 and Ag85A as DNA plus a boost of MVA85A induced a significant increment in the percentage of Ag85A specific CD8^+^ CD107a/b^+^ cells (Figure 3D).

Immune response against *Mtb* is complex, but the participation of macrophages and T lymphocytes in the defense of the host against the pathogen has been extensively demonstrated (Chai et al., 2020). In contrast, the role of humoral immunity in the fight against *Mtb* has been ignored for many years. For this reason, most vaccine candidates focus on the development of appropriate cellular immune responses against *Mtb*. Nevertheless, there are studies demonstrating that polyfunctional and neutralizing antibodies might potentiate humoral effector functions of macrophages and NK cells (Choreño-Parra et al., 2020). By evaluating the production of antibodies, significantly higher levels of anti-Ag85A IgG were detected in mice that received three-doses of Ag85A DNA plus IL-12 DNA as compared to mice immunized with three-doses of Ag85A DNA alone (Figure 2B). Moreover, we observed a significant increase in serum IgG against Ag85A in mice immunized with pCI-Ag85A and MVA85A that also received pCI-IL12 as an adjuvant (Figure 2D). These results are in line with previous reports showing that administration of IL-12 as an adjuvant improves the humoral response of vaccine candidates (McKnight et al., 1994; Germann et al., 1995; Metzger et al., 1996). Importantly, a retrospective analysis in BCG-vaccinated children who received MVA85A showed that elevated anti-Ag85A IgG levels at 28 days after MVA85A boost, reduced the risk of TB development (Fletcher et al., 2016). Therefore an association between the levels of antibodies against Ag85A and the probability of *Mtb* disease in infants was suggested (Fletcher et al., 2016).

Cytokines present in the environment impact on maturation of DCs by modulating their functions during the maturation and migration processes (Banchereau and Steinman, 1998; Fukao et al., 2001). DCs are professional antigen-presentation cells and their main functions are antigen capture and processing, migration to secondary lymphoid organs, and T cell priming. It has been demonstrated that DCs express a single class of high-affinity IL-12R (functionally distinct from those in T and NK cells) that upon binding IL-12 activates members of the NF-κB family and leads to endogenous IL-12 production (Grohmann et al., 1998). Therefore, we hypothesized that in our model, the adjuvant effect of IL-12 DNA on the immunogenicity of the DNA vaccine might be related to the local production of IL-12 by a mechanism similar to the one described by Grohmann et al. In fact, IL-12 produced by IL-12 DNA at the site of injection would induce DCs to secrete more IL-12 eliciting a Th1 profile and activating CD8 cells at the draining lymph node. Furthermore, there is evidence that IL-12 and antigen presentation can be produced by different cells (Bianchi et al., 1999). On the other hand, CDs and monocytes were shown to be capable of producing IFN-γ when cultured in the presence of IL-12, playing an important role in innate immunity and subsequent T helper cell type 1 development *in vivo* (Ohteki et al., 1999).

Taken together, our results demonstrate the ability of IL-12 DNA to improve the humoral and cellular immune responses against Ag85A induced by DNA/MVA85A immunization. Thus, IL-12 DNA might be considered as a tool for designing vaccine strategies to control *Mtb* infection.

## Data Availability Statement

All datasets generated for this study are included in the article/Supplementary Material.

## Ethics Statement

This animal study was reviewed and approved by Operative Unit of the Biological Containment Center from the National Administration of Laboratories and Health Institutes Dr. Carlos G. Malbrán (UOCCCB-ANLIS).

## Author Contributions

The writing—original draft preparation as well as the writing—review and editing of the manuscript was in charge of MPM and VEG. Resources were provided by VEG, MMG, and GC. MPM and MPD were in charge of the development of the methodology. MPM, MPD, and JMP performed the experiments and analysis of the data. NOA and NLT performed formal analyses. VEG was responsible for the supervision, project administration, and conceptualization of the present work. All authors have read and agreed to the published version of the manuscript.

## Conflict of Interest

The authors declare that the research was conducted in the absence of any commercial or financial relationships that could be construed as a potential conflict of interest.

## Footnotes

^1^https://clinicaltrials.gov/ (accessed June 19, 2020).

## Abbreviations

BCG: Bacillus Calmette-Guérin
ConA: Concanavalin A
IL-12: interleukin-12
*Mtb*: *Mycobacterium tuberculosis*
MVA: Modified Vaccinia Ankara
MVA85A: MVA expressing the mycobacterial antigen 85A
pCI: empty pCI plasmid
pCI-Ag85A: pCI plasmid expressing Ag85A
pCI-IL12: pCI plasmid expressing murine IL-12
TB: tuberculosis.
