# Supplementary material for: IL-12 DNA Displays Efficient Adjuvant Effects Improving Immunogenicity of Ag85A in DNA Prime/MVA Boost Immunizations
Source: Front Cell Infect Microbiol. 2020 Sep 23;10:581812. doi: 10.3389/fcimb.2020.581812 (PMC7538621; doi:10.3389/fcimb.2020.581812)
Supplement: Supplementary file 1 [file Data_Sheet_1.PDF]

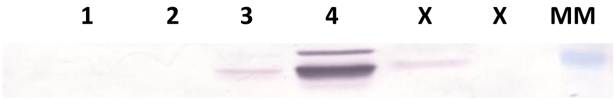

Supplementary Figure S1: pCI-Ag85A and MVA85A express Ag855A. The expression of Ag85A was analyzed by Western blot in total protein extracts from Baby hamster kidney (BHK-21) pCI-Ag85A transfected cells or chicken embryo fibroblasts (CEFs) infected with MVA85A. BHK-21 cells transfected with empty pCI and CEFs infected with wild type MVA were used as negative controls. Western blotting was developed with polyclonal anti-Ag85A antibody. Lanes: 1 – pCI control; 2- MVA control; 3- pCI-Ag85A; 4- MVA85A; MM- Protein Ladder (34 kDa blue band). Lanes X correspond to samples not related to this experiment.
